# Cerebrospinal fluid physiology: visualization of cerebrospinal fluid dynamics using the magnetic resonance imaging Time-Spatial Inversion Pulse method

**DOI:** 10.3325/cmj.2014.55.337

**Published:** 2014-08

**Authors:** Shinya Yamada

**Affiliations:** Division of Neurosurgery, Toshiba Rinkan Hospital, Kanagawa, Japan

## Abstract

Previously there have been no methods for directly tracing the flow of cerebrospinal fluid (CSF) under physiological conditions, and the circulation of CSF has therefore been studied and visualized by injecting a radioactively labeled tracer or contrast medium visible in x-ray images. The newly developed Time-Spatial Inversion Pulse (Time-SLIP) method makes it possible to directly visualize the flow of CSF using magnetic resonance imaging (MRI), permitting CSF dynamics to be depicted in a certain time frame. The CSF dynamics visualized using Time-SLIP has been found to differ markedly from the classical CSF circulation theory described in medical textbooks. It can be said that research on CSF dynamics has advanced to the next stage with the use of this innovative imaging method. Obtaining a more accurate understanding of normal CSF physiology and pathophysiology should lead to improved diagnostic accuracy, permit the identification of new etiological factors in a variety of diseases, and promote the development of new therapeutic approaches.

Descriptions of the physiological circulatory dynamics of cerebrospinal fluid (CSF) can be found mainly in neurosurgery textbooks. More than a hundred years ago, it was only natural that the circulation of CSF was considered to be similar to the circulation of blood. It was naturally thought that the physiology of CSF followed the same pattern as that of blood, which is a typical example of a physiological phenomenon in the body. Harvey Cushing, who was a pioneer in the field of neurosurgery, referred to the CSF as the third circulation (1,2). Since there were previously no methods for directly observing the flow of CSF under physiological conditions, the circulation of CSF has been studied and visualized in humans by injecting a radioactively labeled tracer or contrast medium visible in x-ray images. A needle was inserted into the cerebrospinal space and the observed flow was assumed to reflect that of the CSF. Given this background, the ability to visualize the flow of CSF within a single heartbeat using the phase contrast technique in magnetic resonance imaging (MRI) without the need to inject a tracer into the cerebrospinal space was truly revolutionary. Although considerable research has been conducted using this approach (3-7), it is unfortunately not routinely employed in the clinical setting today. This is because the information obtained using the phase contrast technique is insufficient for making definitive clinical judgments.

Although the newly developed Time-Spatial Inversion Pulse (Time-SLIP) method makes it possible to visualize the flow of CSF using MRI, the imaging procedures and examination time are fundamentally different from those of the phase contrast technique. Therefore, most of the information obtained using these methods cannot be directly compared with each other. In the Time-SLIP method, the CSF itself serves as an endogenous tracer when radiofrequency (RF) pulses are applied. The flow of CSF can be observed for a period of approximately 5 seconds until the effects of the RF pulses diminish and are no longer visible. The Time-SLIP method makes it possible to depict CSF dynamics in a time frame that is not possible with any other method.

The CSF dynamics visualized using Time-SLIP differ markedly from the descriptions of CSF flow that have been given in medical textbooks. CSF is clear like water in the absence of diseases such as meningitis, and it has been demonstrated that it undergoes turnover. However, whether or not the CSF flows like a river from the sites where it is produced to the sites where it is absorbed requires further verification (8,9).

## Visualization of CSF dynamics using MRI with the Time-SLIP method

**Figure 1:**
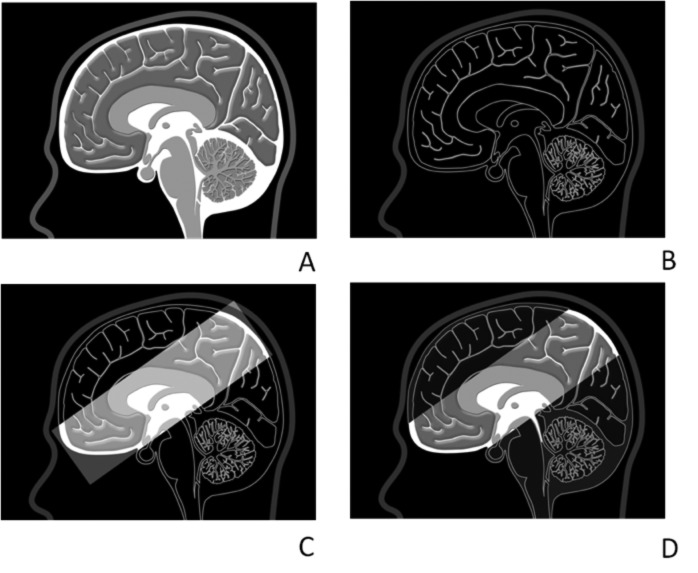
Illustration of the Time-Spatial Inversion Pulse (Time-SLIP) sequence. A non-selective inversion recovery pulse inverts all signals in the field of view from initial longitudinal magnetization (+Mz) to (–Mz) (**A**). Immediately after the initial inversion, a second spatially selective inversion pulse is applied to invert (tag) only the magnetization in the region of interest (white rectangle) (**B**). The magnetization in the marked region is restored to (+Mz), whereas the magnetization elsewhere is (–Mz) (**C**). Images are obtained after a specified period of time. The tagged cerebrospinal fluid (CSF) that has moved into the non-tagged background area produces contrast between the tagged and untagged CSF, which can be visualized during the time period of 1000-5000 ms (arrow) (**D**). Supplementary video 1.

A detailed explanation of the principles of Time-SLIP can be found in other reports (10). Briefly, since the CSF itself is marked with RF pulses in MRI, the dynamics of the CSF can be visualized as long as this marking persists. It takes about 8 seconds for signals of marked water to return to their original levels in a 1.5-tesla magnetic field. However, the practical observation time is considered to be just under 5 to 6 seconds, since visualization is based on the contrast between the marked CSF and background area. In the Time-SLIP method, a non-selective inversion recovery (IR) pulse is applied to the entire field of view, a selective inversion pulse is applied to the region to be examined, and images are then acquired after a specified delay time (Figure 1). The delay time for image acquisition can be adjusted to acquire images at different timings. The images can be acquired one by one and then arranged in sequence on the time axis or can be acquired as fully sequential images. Images acquired using the former method tend to be of higher quality in terms of resolution. However, due to the principles of this method, the continuity of the images is lost when they are displayed as a movie in the situation when there is more than one force driving CSF (11).

The fact that CSF motion varies in response to cardiac pulsation and respiratory motion can be observed as a fluid level fluctuation in the ventricular external drainage tube from the patient cerebral ventricle, observing the changes in fluid level of the CSF during surgery and the lumbar puncture. In other words, CSF dynamics exhibit various types of motion according to the combination of cardiac pulsation and respiratory motion, which means that the data must be acquired in real time (11). This driving force is also an issue in the phase contrast technique. In the conventional phase contrast technique, images are acquired by adding and averaging images for many cardiac cycles using the cardiac pulsation as a trigger, and the motion of CSF attributable to respiratory motion is not taken into consideration (5,7). During surgery and in scans with Time-SLIP, the flow of CSF attributable to respiratory motion is observed, and it has been found to have a greater effect than the flow attributable to cardiac pulsation (11). Considering this point, the variation among data sets measured using the phase contrast technique, which has been assumed to be a problem, may in fact be due to the CSF motion attributable to respiratory motion. Image acquisition in the phase contrast technique usually takes about 2 to 3 minutes. It is important not only to look at the results, but also to understand the principles of the technique and the process by which the results are obtained.

## Physiological CSF dynamics in the ventricular system

**Figure 2:**
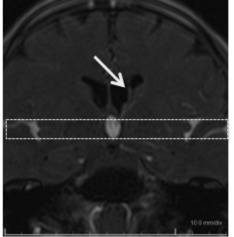
The flow of cerebrospinal fluid (CSF) into the lateral ventricles is depicted (arrow) when radiofrequency (RF) pulses are applied to the CSF in the third ventricle (dotted rectangle) in this coronal view of the normal brain. Supplementary video 2.

Using Time-SLIP, the flow of CSF from the third ventricle into the lateral ventricles has been observed (Figure 2). CSF flow into the lateral ventricles is seen when RF pulses are applied to the CSF in the third ventricle (a process referred to as tagging). Since the flow of CSF had previously been assumed to be from the lateral ventricles to the third ventricle based on the descriptions in medical textbooks, it took some time to understand this finding. This flow is opposite to the conventional concept of CSF physiology and can be described as a backflow into the lateral ventricles.

**Figure 3:**
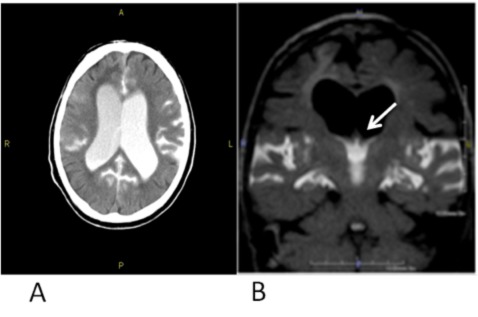
Patient with communicating hydrocephalus. Metrizamide CT cisternography indicates ventricular reflux (black arrow) (**A**). However, cerebrospinal fluid (CSF) flow into the lateral ventricles from the third ventricle is not observed using the Time-SLIP method (white arrow) in the hydrocephalic brain (**B**). Supplementary video 3.

Actually, it was previously thought that this type of CSF flow does not occur in healthy persons (12), but only in patients with hydrocephalus, who have impaired CSF circulation (13-15). This was based on the finding that when contrast medium or radioisotope (RI) is injected into the CSF in the lumbar subarachnoid space in patients with hydrocephalus, the CSF is seen to flow back into the lateral ventricles. This finding, which is called ventricular reflux of the CSF, has been used to confirm the diagnosis of hydrocephalus (13-15) (Figure 3A).

When we consider this finding more deeply, it is clear that we are unable to answer the simple question “Where is the CSF produced in the case of hydrocephalus in which ventricular reflux is observed?” Nevertheless, this theory was widely accepted as the truth for nearly 50 years until CSF dynamics could be clearly visualized using Time-SLIP. No CSF reflux from the third ventricle to the lateral ventricles was observed in a patient with hydrocephalus using Time-SLIP MR imaging (Figure 3B). Since stagnation of the CSF occurs due to impaired CSF circulation in patients with hydrocephalus, a tracer such as contrast medium or RI reaches the lateral ventricles by the mixing and diffusion that accompanies the agitation after the injection into the lumbar subarachnoid space. We can now appreciate that what is actually observed is this phenomenon. In other words, it can be said that these exogenous tracer studies (13-15) are able to demonstrate the presence of communication between the site of injection and the final destination, but it cannot be said that such studies are able to trace the bulk flow of CSF.

**Figure 4:**
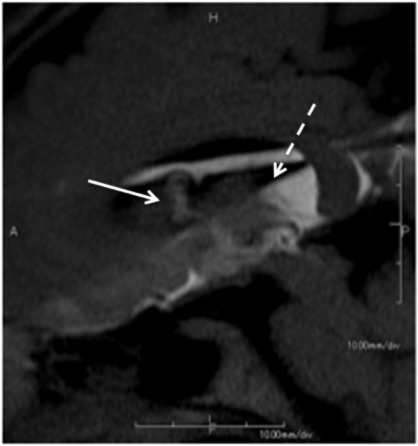
Sagittal oblique view of the normal brain. Pulsatile motion through the aqueduct is observed. Cerebrospinal fluid (CSF) reflux from the third ventricle to the lateral ventricle is also observed. However, no CSF motion is seen in the body of lateral ventricle (dotted arrow). Supplementary video 4.

The CSF motion visualized using Time-SLIP does not show unidirectional backflow of CSF into the lateral ventricles, but rather demonstrates that the CSF in the lateral ventricles and the CSF in the third ventricle are actively exchanged through the foramen of Monro in the normal brain. On the other hand, there is virtually no flow (or only extremely slow flow) of CSF in the body of the lateral ventricles, except in the area adjacent to the foramen of Monro (Figure 4). At least in humans, the steady state flow from posterior to anterior observed in recent exogenous tracer experiments in animals is not seen (16). It can be concluded that the injection of exogenous tracers is unable to accurately show the flow of CSF and that the CSF flow observed is obviously artificially induced flow.

**Figure 5:**
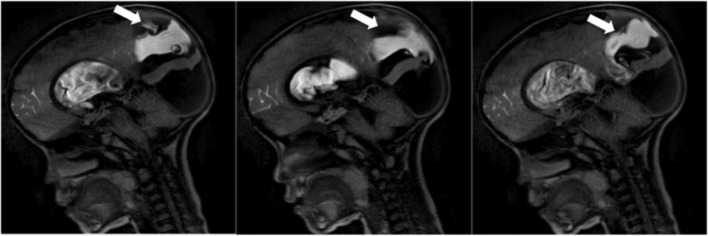
Seven-year-old boy who presented with ventriculomegaly associated with aqueductal stenosis. He moved his head during the scan. The cerebrospinal fluid (CSF) in the body of the lateral ventricle was agitated by head movement (arrows). Supplementary video 5.

In examinees who are unable to remain still and therefore move their heads during MRI scanning, the CSF in the body of the lateral ventricles is strongly agitated (Figure 5). Considering this point in greater detail, it should also be noted that almost all previous research on CSF physiology was limited to observation of the CSF in anesthetized animals or examinees who remained still during MRI scanning. However, since people and animals do not remain still all day long, the motion of CSF that is induced by walking, sitting, or running should be considered to more accurately reflect true physiological conditions.

The circulation of CSF is clearly not like that of blood, which flows through tubular blood vessels at high speed. A more accurate model may be that of placing the brain and spinal cord in a glass container, immersing them in water, and then shaking the container. Even though CSF motion can only be visualized in real time for a period of several seconds, it is clear that these observations are totally different from the classical concept of CSF circulation.

## Possible paracrine functions mediated by the CSF in the ventricular system

**Figure 6:**
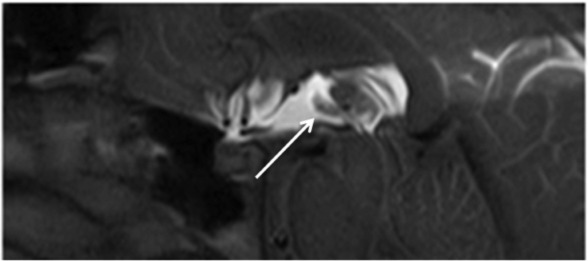
Midsagittal view of a normal volunteer showing turbulent cerebrospinal fluid flow in the third ventricle with the head stationary (arrow). Supplementary video 6.

CSF motion in the third and fourth ventricles is swirling vortex-type flow even when the head is stationary (Figure 6). The area around the third ventricle contains a dense arrangement of vital structures that are related to the circadian rhythm. It has recently become possible to directly observe the inner surface of the ventricles by opening a small hole in the brain and inserting an endoscope (17). Due to remarkable improvements in spatial resolution, the visualization capabilities are completely different from those in the past. It is now possible to insert an endoscope into the third ventricle by advancing it from the lateral ventricle through the foramen of Monro after opening a small hole in the brain. We can see capillaries running from the pituitary gland in the anterior direction. Exposed red-colored blood vessels can be seen in the wall of the third ventricle. Although this may be a rather unfamiliar concept, a new theory of volume transmission involving hormonal transmitters (eg, orexin, prostaglandin D) has been proposed in contrast to the so-called neurotransmission in the form of synaptic transmission (18-21). This involves hormonal transmission, which functions in a manner similar to internal secretion in the blood. Volume transmission is thought to transmit signals to surrounding tissues by means of hormonal transmitters (ie, paracrine system in the central nervous system). The mechanism by which the CSF transports hormonal transmitters and allows their interaction via the CSF is referred to as CSF signaling (20). This mechanism transmits signals over a considerable distance, for example from the pineal body to the pituitary gland. The turbulence and swirling CSF flow in the third ventricle (10), should have some functional significance in terms of CSF signaling, which can be referred to as the CSF paracrine system.

We can easily imagine that the function of the CSF is surely not only to protect the brain from external forces (because the weight of the brain itself is offset as a result of being suspended in water, in accordance with Archimedes’ principle) or to serve as a means for eliminating wastes from the brain (22-26) although this may not be the textbook explanation in the field of CSF physiology. Here, it is a means for disposing of waste matter from the brain.

## Are the arachnoid villi a major site of CSF absorption in humans?

The standard method for determining the amount of CSF is based on an ingenious ventriculo-cisternal perfusion experiment described by Pappenheimer et al (27,28). Artificial CSF containing a known concentration of a substance is injected and then collected after dilution. The total amount of CSF can be calculated from the concentration of the substance in the collected fluid. Currently, CSF production levels in humans and animals are measured using this method (27,29-33). However, if you carefully check the calculation formula (27), you can see that it is based on the assumption that the CSF in the ventricular system is not absorbed by brain tissue. With the exception of some studies (8,9,34,35), most studies on CSF physiology have not considered the relationships with brain interstitial fluid.

Brain interstitial fluid has the same composition as CSF, and there is no barrier between them like the blood-brain barrier. Normally, free exchange occurs between the CSF and brain interstitial fluid (35,36) through the Virchow-Robin spaces (ie, perivascular spaces) (34,37,38), which pose the least resistance to flow. Although the blood-brain barrier is known to have diverse and complex functions that enable selective passage of high-molecular weight substances, water itself freely passes through the blood-brain barrier. Hence, it can be concluded that at least a portion of the CSF is composed of water that has passed through the blood-brain barrier (8,9,39).

According to medical textbooks, the CSF is produced by the choroid plexus. But this raises an important question: When the cerebral aqueduct or the outlets of the fourth ventricle are obstructed, where is the CSF downstream from the point of occlusion produced? This is another example of a situation in which the CSF physiology described in current medical textbooks is unable to fully explain CSF flow under physiological or pathological conditions.

At present, Time-SLIP is unable to depict the correlated dynamics of the CSF and brain interstitial fluid. Although this may be a bit of a digression from the main topic, which is the visualization of CSF using Time-SLIP, the production and absorption of CSF mediated by brain capillaries is one of the most important issues in the reassessment of CSF physiology (9,39). This has been pointed out as a matter of fact since the very early stages of CSF research, and some studies have been conducted since that time (12,39-43). However, the importance of this absorption pathway of CSF is still not adequately conveyed in standard medical textbooks, also CSF drainage from central nervous system occurs via the lymphatic system (38,44-47). It is clear that there are still many questions regarding the absorption pathways of CSF.

**Figure 7:**
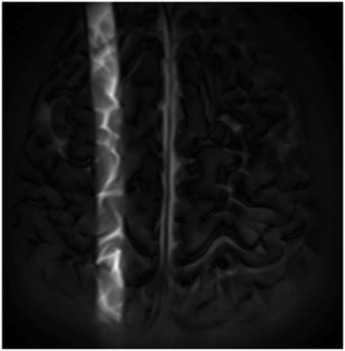
Axial view of the cerebral convexity in a normal volunteer. No cerebrospinal fluid (CSF) motion or flow is observed over the cerebral convexity. Supplementary video 7.

Time-SLIP allows the dynamics of CSF to be depicted under the most physiological conditions because it does not require the injection of a tracer. The CSF itself serves as an endogenous tracer. The most important finding related to the absorption pathways of CSF obtained using Time-SLIP is that no flow or pulsation of the CSF is observed over the cerebral convexity (Figure 7). Even though strong CSF pulsation is observed from the prepontine and basilar cisterns to the Sylvian fissure, neither continuous flow nor pulsation of the CSF is observed from the Sylvian fissure to the cerebral fornix. The superficial Sylvian vein is present at this site, and the arachnoid membrane that covers it is strongly adherent to the vein. This indicates that the Sylvian fissure is practically at the distal end of the subarachnoid space. Although CSF is present on the brain surface, the Sylvian fissure poses considerable resistance to the flow of CSF. Accordingly, the absorption pathway of CSF is thought to exist somewhere proximal to the Sylvian fissure. As mentioned above, the most likely candidate is the route by which macromolecules in the CSF are absorbed into the lymphatic system from areas surrounding each of the cranial nerves or spinal nerves.

## CSF motion in the spinal subarachnoid space

When the flow of CSF in the subarachnoid space of the spine is visualized, the CSF is seen to pulsate along the ventral side of the spine in the supine position, but hardly any CSF flow is seen along the dorsal side of the spine. However, the CSF begins to pulsate along the dorsal side of the spine when the same examinee is placed in the prone position (10). On the basis of this finding as well, it appears that the CSF simply starts to move toward the location where it can flow more easily with little resistance (10). This observation differs considerably from the standard textbook description, which states that the CSF flows downward on the dorsal side of the spine and upward on the ventral side of the spine.

The spinal subarachnoid space is a cylindrical structure with its tip at the caudal end and it does not contain any internal partitions. It is very difficult to imagine that CSF flows in opposite directions on the dorsal side and ventral side. The observations underlying this theory of CSF flow in the subarachnoid space were reported by Di Chiro (48), who surgically removed the vertebral arches of an animal in the prone position and injected dye into the ventricles. Since the dye was seen to move downward in the spinal subarachnoid space, he concluded that the CSF flows downward on the dorsal side of the spine. In addition, since radioisotope injected into the lumbar subarachnoid space enters the cranium in humans (12), it was assumed that the CSF must flow upward on the ventral side of the spine because it flows downward on the dorsal side. Now that Time-SLIP has provided new information on CSF dynamics (10,11), it is clear that this assumption was a complete misinterpretation based on an experiment using an exogenous tracer. However, it has been accepted as the truth in CSF physiology for many years, and even today.

## Does the CSF circulate?

**Figure 8:**
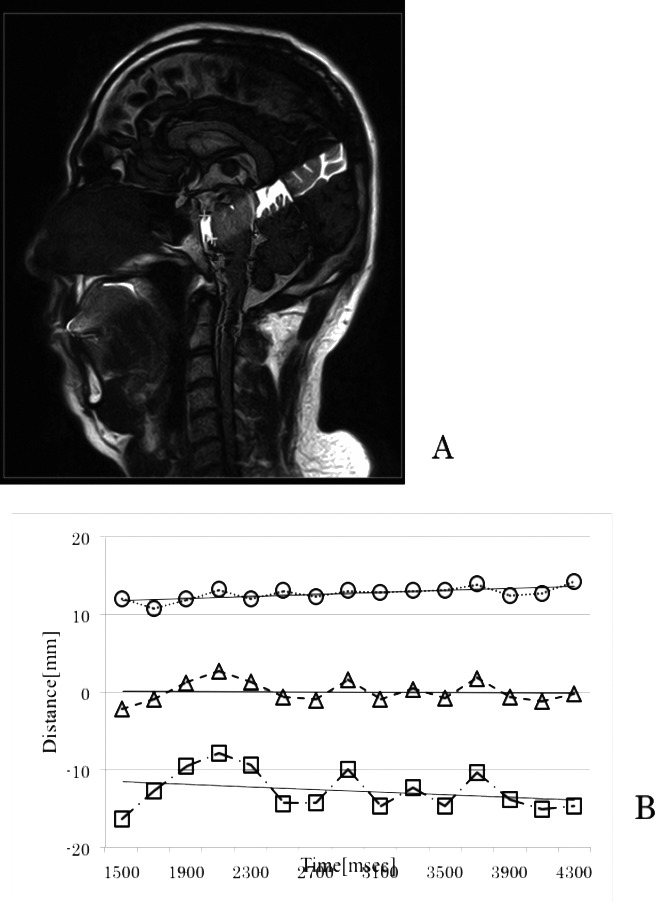
Cerebrospinal fluid (CSF) motion in the prepontine cistern was traced using semi-automated tracing software (Dynatracer) in the normal brain. The open circles and open squares indicate the upper and lower positions of the tagged CSF. The open triangles indicate the average of the upper and lower points. The tagged CSF had spread out by the time of observation. However, no unidirectional bulk CSF flow was observed.

**Figure 9:**
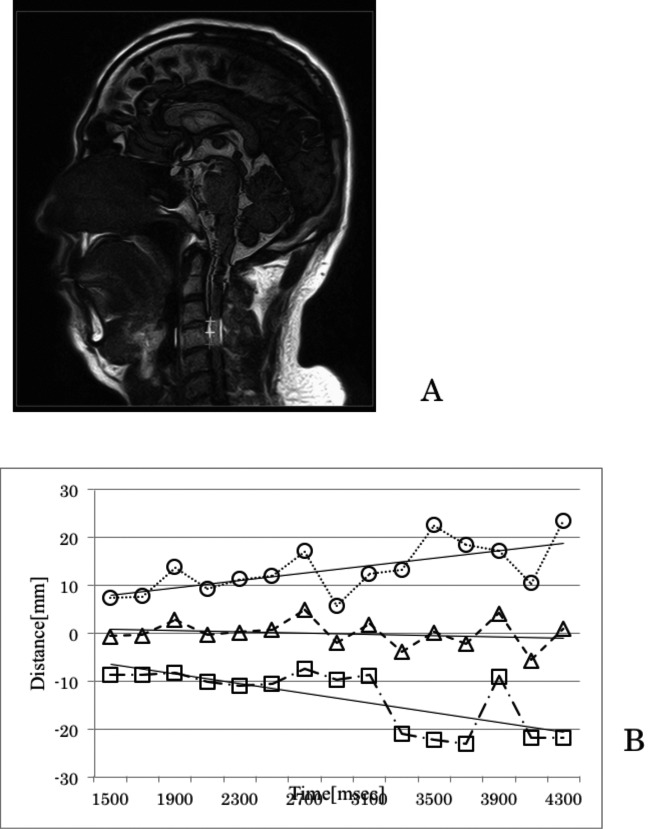
Cerebrospinal fluid (CSF) motion in the spinal subarachnoid space was traced using semi-automated tracing software (Dynatracer) in a normal volunteer. The open circles and open squares indicate the upper and lower positions of the tagged CSF. The open triangles indicate the average of the upper and lower points. The tagged CSF had spread out by the time of observation. However, no unidirectional bulk CSF flow was observed.

**Figure 10:**
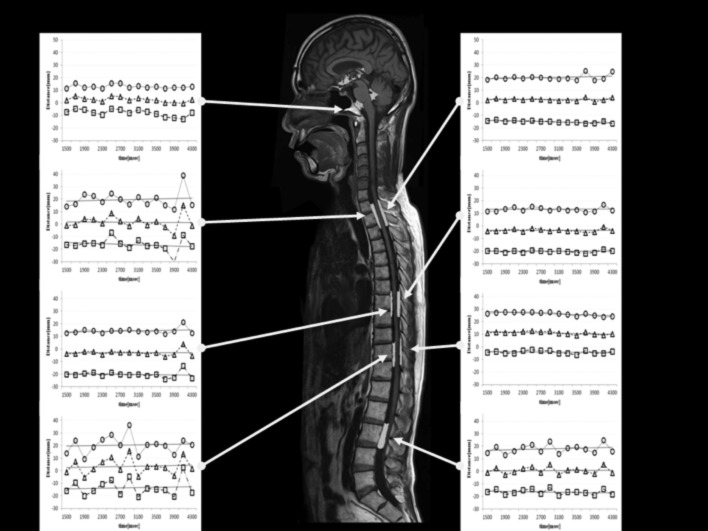
Midsagittal view of the entire brain and spine showing labeled cerebrospinal fluid (CSF) motion at different locations in the subarachnoid space as well as in the ventricles. The labeled CSF tended to spread over time, but no unidirectional bulk CSF flow was observed. Supplementary video 8.

CSF pulsation observed by Time-SLIP was traced using semi-automated tracing software called Dynatracer (49). The movement of the tagged CSF can be traced over an observation time of 5 seconds. In Figures 8 to 10, the x-axis of the graphs represents the time after tagging and the y-axis represents the distance of CSF movement. The open circles and open squares on the graphs indicate the upper and lower positions of the tagged CSF over time. The open triangles indicate the average of the upper and lower points. These points tend to spread out over the time, but no unidirectional CSF flow was observed in the prepontine cistern (Figure 8) or in the spinal subarachnoid space (Figure 9). The CSF was found to exhibit pulsatile movement, but no bulk flow. These results suggest that there is no CSF circulation (bulk flow) as seen in the blood. In other words, CSF does not flow from the site of production to the site of absorption (Figure 10). This finding of a lack of CSF circulation does not conflict with the CSF theory recently proposed by Klarica and Oreskovic (50,51). In their theory, the cerebral blood vessels are responsible for CSF (water) transport, so there should be no CSF bulk flow from the choroid plexus in the lateral ventricles to the arachnoid villi on the cerebral convexity (8,9,52).

## Conclusion

It can be said that research on CSF dynamics has advanced to the next stage with the use of the innovative imaging method known as Time-SLIP. Obtaining a more accurate understanding of normal CSF physiology and pathophysiology should lead to improved diagnostic accuracy, permit the identification of new etiological factors in a variety of diseases, and promote the development of new therapeutic approaches.
